# Microbial neuraminidase induces TLR4-dependent long-term immune priming in the brain

**DOI:** 10.3389/fncel.2022.945229

**Published:** 2022-07-28

**Authors:** María del Mar Fernández-Arjona, Ana León-Rodríguez, Jesús M. Grondona, María Dolores López-Ávalos

**Affiliations:** ^1^Laboratorio de Medicina Regenerativa, Grupo de investigación en Neuropsicofarmacología, Hospital Regional Universitario de Málaga, Málaga, Spain; ^2^Instituto de Investigación Biomédica de Málaga-IBIMA, Málaga, Spain; ^3^Laboratorio de Fisiología Animal, Departamento de Biología Celular, Genética y Fisiología, Facultad de Ciencias, Universidad de Málaga, Málaga, Spain

**Keywords:** microglia, neuraminidase, neuroinflammation, TLR2, TLR4, mouse, immune priming

## Abstract

Innate immune memory explains the plasticity of immune responses after repeated immune stimulation, leading to either enhanced or suppressed immune responses. This process has been extensively reported in peripheral immune cells and also, although modestly, in the brain. Here we explored two relevant aspects of brain immune priming: its persistence over time and its dependence on TLR receptors. For this purpose, we used an experimental paradigm consisting in applying two inflammatory stimuli three months apart. Wild type, toll-like receptor (TLR) 4 and TLR2 mutant strains were used. The priming stimulus was the intracerebroventricular injection of neuraminidase (an enzyme that is present in various pathogens able to provoke brain infections), which triggers an acute inflammatory process in the brain. The second stimulus was the intraperitoneal injection of lipopolysaccharide (a TLR4 ligand) or Pam3CSK4 (a TLR2 ligand). One day after the second inflammatory challenge the immune response in the brain was examined. In wild type mice, microglial and astroglial density, as well as the expression of 4 out of 5 pro-inflammatory genes studied (TNFα, IL1β, Gal-3, and NLRP3), were increased in mice that received the double stimulus compared to those exposed only to the second one, which were initially injected with saline instead of neuraminidase. Such enhanced response suggests immune training in the brain, which lasts at least 3 months. On the other hand, TLR2 mutants under the same experimental design displayed an enhanced immune response quite similar to that of wild type mice. However, in TLR4 mutant mice the response after the second immune challenge was largely dampened, indicating the pivotal role of this receptor in the establishment of immune priming. Our results demonstrate that neuraminidase-induced inflammation primes an enhanced immune response in the brain to a subsequent immune challenge, immune training that endures and that is largely dependent on TLR4 receptor.

## Introduction

During recent years neuroinflammation is attracting attention considerably because it is suspected to be underneath a variety of neurological disturbances, including behavioral alterations and neurodegeneration (Amor et al., 2014; Ransohoff, 2016). Factors triggering neuroinflammation are diverse and can be exogenous (pathogens, traumatisms, toxins, etc.) or endogenous (misfolded protein accumulation, stroke, etc.). Peripheral inflammation may also translate into the central nervous system (CNS), as some immune mediators are able to gain access to the nervous tissue (Godbout et al., 2005; Henry et al., 2008; Lu et al., 2009). In many cases neuroinflammation is a severe and acute process, aimed to first eliminate an infectious agent or tissue debris derived from injury, and later repair the nervous tissue (O'Callaghan et al., 2008). In such process a variety of elements are called into action, most notably peripheral immune cells (a prominent infiltration of lymphocytes, monocytes and macrophages occur) and resident glial cells (microglia and astrocytes) (Graeber, 2014). These cells get activated and produce and secrete high amounts of inflammatory mediators, cytokines and chemokines. After peaking, neuroinflammation usually declines slowly toward the basal situation existing prior to the immune challenge, with the withdrawal of peripheral immune cells and the decline in levels of inflammatory mediators. Resident cells, microglia and astrocytes, also deactivate, although more slowly (Ransohoff et al., 2015). However, a single inflammatory event may prime the response to future immune challenges. Thus, immune memory of previous events drives plastic immune responses to future challenges, response that can be enhanced, a so called immune training or priming, or suppressed, referred to as immune tolerance (Natoli and Ostuni, 2019). Both aspects of immune memory have been well described and broadly studied in the peripheral immune system, as they are involved in relevant issues such as vaccination, graft tolerance/rejection and tumor progression, among others (Simpson and Marshall, 2001; Dumauthioz et al., 2018; Freise et al., 2019). However, about immune memory within the CNS evidences exists (Nott and Glass, 2018; Neher and Cunningham, 2019; Lebrun et al., 2020), but it has not been so widely studied. Particular interest has been raised by the priming of microglial cells, which are regarded as orchestrators of immune responses within the brain (Kettenmann et al., 2011; Sierra et al., 2016; Wolf et al., 2017). Primed microglial cells retain an immune training that makes them undergo exacerbated responses to new stimuli, even these being mild or of a different nature, and either of central or peripheral origin (Godbout et al., 2005; Purisai et al., 2007; Norden et al., 2015; Martins-Ferreira et al., 2021). Interestingly, primed microglia has been shown to be a risk factor for neurodegeneration, behavioral alterations and even psychiatric disorders (Perry and Holmes, 2014; Colonna and Butovsky, 2017; Bartels et al., 2020; Martins-Ferreira et al., 2021; Muzio et al., 2021).

As main sensors of the nervous tissue milieu and first line of defense against exogenous threats (such as viruses and bacteria), immune cells are endowed with an ample array of pattern recognition receptors (PRRs) which are involved in detecting both endogenous molecular patterns (like alarmins) and infectious agents (Gordon, 2002; Takeuchi and Akira, 2010). Within the growing family of PRRs, toll-like receptors (TLRs) were the first to be identified, and are the most widely studied (Akira et al., 2006; Kumar et al., 2011). Among the various subtypes of TLRs described so far, TLR2 and TLR4 are relevant in the initiation of the inflammatory response within the CNS (Kawai and Akira, 2006; Lehnardt, 2010). Moreover, TLR4- signaling in microglial cells plays an important role in maintaining homeostasis within the brain (Milanski et al., 2009; Masson et al., 2015; Reis et al., 2015). Thus, although TRL2 and TLR4 are likely candidates to mediate in the establishment of brain immune memory, information about this possibility is quite limited (Weber et al., 2013; Facci et al., 2014).

Therefore, here we sought to explore (1) if immune priming is stablished in the brain when exposed to an immune challenge, (2) if it remains for a long period of time, and (3) the relevance of TLR receptors −2 and −4 in such process. A double stimulus paradigm was design, where the first stimulus was aimed to prime the immune memory, and the second one served to measure the amplitude of the immune response and thus evidence if immune priming was in fact established. The priming stimulus was a single intracerebroventricular (ICV) injection of the microbial enzyme neuraminidase (NA), which is part of pathogens (virus and bacteria) able to provoke infections in the brain (O'Toole et al., 1971; Love et al., 1985; Steininger et al., 2003; Finsterer and Hess, 2007; Yildizdas et al., 2011; Lewis and Lewis, 2012). Thus, the ICV-administration of NA represents a model of acute neuroinflammation triggered by a microbial factor, although considered sterile because no pathogen is present. This model has been extensively used and described by our group (Grondona et al., 1996; Gomez-Roldan et al., 2008; Granados-Duran et al., 2015; Fernandez-Arjona et al., 2017). To evaluate the persistence of the immune priming over time, the second stimulus was applied 3 months after NA. This second immune challenge was systemic, and consisted in the intraperitoneal (IP) injection of well-known ligands of TLR2 (Gambhir et al., 2012) and TLR4 (Beutler, 2000) receptors. The immune response within the brain after this second challenge was evaluated to evidence a plausible immune priming. With the purpose of investigating the implication of TLR2 and TLR4 on the generation of immune priming status, mutant strains of each receptor were included in our study.

## Materials and Methods

### Animals

TLR2 (B6.129-Tlr2tm1kir/J) and TLR4 (B6.B10ScN-Tlr4lps-del/JthJ)-deficient mice (TLR2^−/−^ and TLR4^−/−^ respectively) were bred by The Jackson Laboratory and purchased through Charles River Laboratories (Lyon, France). The wild-type strain (WT) used was C57BL/6 J. These animals were maintained in the animal house at Universidad de Málaga, under a 12 h light/dark cycle, at 23 °C and 60% humidity, with food and water available *ad libitum*. Animal care and handling were performed according to the guidelines established by Spanish legislation (RD 53/2013) and the European Union regulation (2010/63/EU). All procedures performed were approved by the ethics committee of Universidad de Málaga (Comité Ético de Experimentación de la Universidad de Málaga; reference 2012-0013). All efforts were made to minimize the number of animals used and their suffering.

The experimental design carried out with WT, TLR2^−/−^ and TLR4^−/−^ mice consisted in two inflammatory stimuli applied 3 months apart. The first stimulus was the intracerebroventricular (ICV) injection of neuraminidase; control animals were injected with saline. Three months later, a peripheral stimulus was applied, consisting in an intraperitoneal (IP) injection of lipopolysaccharide (LPS) or Pam3CSK4 (P3C; see below); sham mice were injected with saline. Mice were sacrificed 24 h later. Details of ICV and IP injections are explained next.

### Intracerebroventricular and intraperitoneal injections

An acute neuroinflammatory process was generated in mice by a single injection of the enzyme neuraminidase (NA) within the right lateral ventricle of the brain (Fernandez-Arjona et al., 2019a). Sham animals were injected with 0.9% sterile saline. The ICV injection procedure was performed as previously were described (Fernandez-Arjona et al., 2019a). Briefly, the animals were anesthetized with ketamine/xylazine solution (80 and 12 mg/kg, respectively; Sigma-Aldrich) and positioned in a stereotaxic apparatus. A scalp incision along the sagittal midline was performed to access the skull and the bone was perforated with a drill in the following coordinates: 0.1 mm posterior and 0.9 mm lateral from Bregma (Franklin and Paxinos, 2008). NA from *Clostridium perfringens* (Sigma-Aldrich, N3001) dissolved in 0.9% sterile saline was administered by a single injection 2.0 mm below the dura mater with the aid of a pump (KDS-210-CE, KD Scientific); the amount of NA injected was 75 mU (1 μL), perfused for 5 min at a rate of 0.2 μL/min. This dose was based on our previous experience with rats and mice (Granados-Duran et al., 2015).

The second inflammatory stimulus consisted of an IP injection 3 months after the ICV. TLR4^−/−^mice were injected with the synthetic triacylated lipoprotein Pam3CSK4 (P3C; InvivoGen, 12A10-MM, 5 mg/kg), which is an agonist of the receptor TLR2 (Kumar et al., 2011). In the case of the TLR2^−/−^ strain, ultrapure LPS (a highly purified fraction from *E. coli*) was used (InvivoGen, 13I06-MM, 300 μg/kg), which is a specific ligand of TLR4 (Kawai and Akira, 2006). Sham animals were IP injected with 0.9% sterile saline. Mice were sacrificed 24h after the IP injection by cardiac perfusion of heparinized sterile saline. The brains were extracted and divided by the sagittal line into two halves, intended for RNA extraction and immunohistochemistry respectively. A total of 3–4 animals were included in each group of ICV saline injected controls, and 5–7 mice in groups ICV injected with NA.

### Immunohistochemistry

Free-floating vibratome sections were obtained in coronal plane (40 microns thick). Sections containing the hypothalamus (distance from Bregma −0.5 to −1.5 mm) were immunostained. First, they were treated with 10% methanol and 3% hydrogen peroxide in PBS during 45 min to quench endogenous peroxidase. After washings with PBS, nonspecific binding sites were saturated with PBT solution (0.3 % bovine serum albumin, 0.3% Triton X-100 in PBS pH 7.3). Primary antibodies were rabbit anti-rat IBA1 (1:500, Wako #019-19741) and anti-rabbit GFAP (1:1000, Sigma-Aldrich #G9269), both diluted in PBT solution and incubated overnight at 4 °C. The following morning the sections were washed with PBS and incubated with the biotinylated secondary antibody (goat anti-rabbit IgG from Pierce #31820 and goat anti-rat IgG from Invitrogen #31830) diluted 1:1000 in PBT, at room temperature for 1.5 h. The avidin-biotin-complex amplification system (ABC; 1:250; Thermo Fisher Scientific #32020) was used afterwards (at room temperature, 45 min) to detect the secondary biotinylated antibody. The peroxidase activity was revealed with 0.05% diaminobenzidine and 0.03% hydrogen peroxide in PBS for 10 min. After thorough washes, the sections were then mounted onto gelatin-coated slides and air-dried. Afterwards they were dehydrated in graded ethanol, cleared in xylene, and coverslipped with Eukitt mounting medium.

### Image acquisition and quantitative analysis

IBA1- or GFAP-positive cell counts were performed in the medio-dorsal periventricular region of the hypothalamus. For this purpose, digital color images of DAB-stained tissue sections were obtained using an Olympus VS120 microscope at 40x UPLSAPO objective. The areas of interest were scanned in at least two sections per animal. IBA1 or GFAP immunoreactive cells were manually counted, and referred to the area sampled (positive cells / mm^2^).

### RNA isolation and RT-qPCR analysis

From the left side of the brains, the hypothalamus was dissected out under a stereo microscope and RNase-free conditions. Immediately after dissection, tissue samples were quickly frozen with dry ice and stored at −80 °C. Total RNA was later isolated using TRIzol reagent (Invitrogen). One milliliter of reagent was added to about 100 mg of tissue, and RNA was isolated following manufacturer's instructions. RNA was dissolved in ≤ 50 μL of RNase-free water, and its concentration was measured in a NanoDrop microvolume spectrophotometer (NanoDrop 1000, Thermo Fisher Scientific). The A260/280 ratio of the isolated RNA was usually about 1.8.

cDNA synthesis was performed using the SuperScriptTM III First-Strand Synthesis (Invitrogen). The reaction mix was prepared according to the manufacturer's protocol. RNA was added in order to have 500 ng in a reaction volume of 20 μL, therefore the resulting cDNA concentration would be equivalent to 25 ng RNA/μL. cDNA was stored at −20 °C.

Real-time PCR (qPCR) was used to quantify specific mRNAs represented in cDNA samples. The hot start reaction mix FastStart Essential DNA Green Master (Roche), based on SYBR Green I fluorescence dye, was used for this purpose. PCR reactions were prepared following manufacturer's instructions. Forward and reverse primers were used at a final concentration of 0.4 μM. The amount of cDNA added was equivalent to 10 ng RNA (amount established after preliminary trials). The qPCR reaction was carried out in a LightCycler 96 Instrument (Roche). The information obtained (amplification curves, melting curves and crossing points, CP, or cycle threshold, Ct) for each transcript was processed using the software provided with the LightCycler equipment. The relative expression of each target gene to the reference gene glyceraldehyde 3-phosphate dehydrogenase (GAPDH) was calculated by the 2^−ΔΔCt^ method (Livak and Schmittgen, 2001). Primers to target the mRNA of genes related to inflammation (Table 1) were designed using the program Primer3 (https://primer3.org/). Target genes sequences were obtained from the Genbank NCBI Reference Sequence (https://www.ncbi.nlm.nih.gov/).

**Table 1:** Sequences of the primers used for qPCR.

**Gene ID**	**Accession #**	**Primers sequence (5'**→**3')**		**Amplicon (bp)**
		**Forward**	**Reverse**
*TNFα*	NM_013693.3	GATTATGGCTCAGGGTCCAA	ACAGAGGCAACCTGACCACT	197
*IL1β*	NM_008361.4	GAGTGTGGATCCCAAGCAAT	ACGGATTCCATGGTGAAGTC	201
*Gal-3*	NM_001145953.1	TAATCAGGTGAGCGGCACAG	CGGATATCCTTGAGGGTTTGG	107
*NLRP3*	NM_145827.4	GCTAAGAAGGACCAGCCAGA	CAGCAAACCCATCCACTCTT	99
*MHC II*	NM_010378.3	TCCTCAAGCGACTGTGTTCC	CGTCTGCGACTGACTTGCTA	134
*Gapdh*	NM_008084.3	TGAACGGGAAGCTCACTGG	TCCACCACCCTGTTGCTGTA	307

### Statistical analysis

Statistical analysis was carried out using SPSS Statistics software. The Kolmogorov-Smirnov normality tests, along with the Levene homoscedasticity test, were used to verify if data could be analyzed by parametric methods. Three-way analysis of variance (ANOVA) was used to compare groups' means. “ICV injection” was the first factor, which had two levels: NA and saline. “IP injection” was the second factor, with three levels: saline, LPS and P3C. Finally, “genotype” was the third factor, which was split into three levels: wild type, TLR2^−/−^ and TLR4^−/−^ mouse strains. Multiple comparisons were performed by Tukey's test, and the differences were considered significant when a *P* < 0.05 was obtained.

## Results

### Enhanced microgliosis upon a second inflammatory stimulus in WT and TLR2^–/–^ mice, but not in TLR4^–/–^ mice

**Figure 1:**
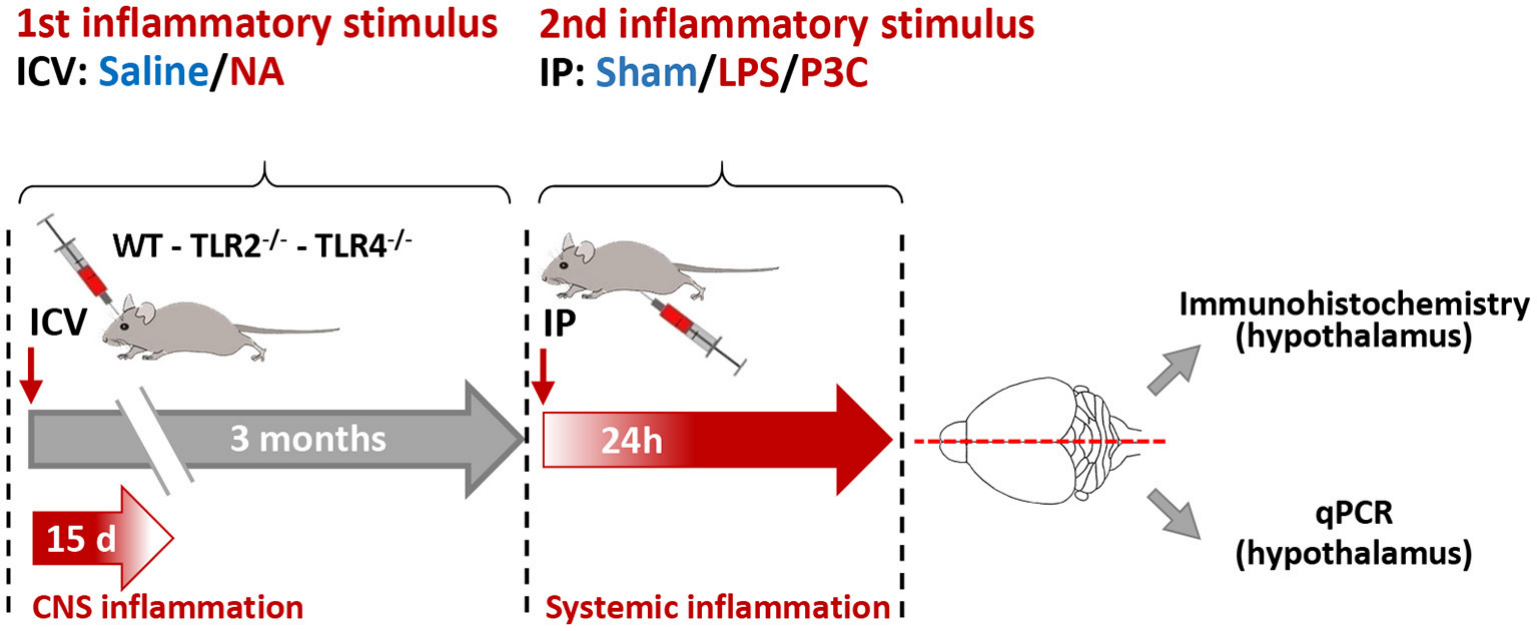
Scheme of the experimental design. Wild type (WT), TLR2 and TLR4 mutant mouse strains were subjected to a first inflammatory stimulus consisting of an ICV injection of neuraminidase (NA) or saline (as control). NA-induced inflammation lasts about 15 days. Three months later a second inflammatory stimulus was applied, consisting of an intraperitoneal (IP) injection of LPS or P3C; saline was injected to control animals (sham). Twenty-four hours after this second stimulus the animals were anesthetized, perfused with saline and their brains removed. The brains were divided by the mid-sagittal line; the right half was used for immunohistochemistry, and the left half was used for RNA isolation and gene expression quantification by qPCR. These studies focused on the hypothalamus.

**Figure 2:**
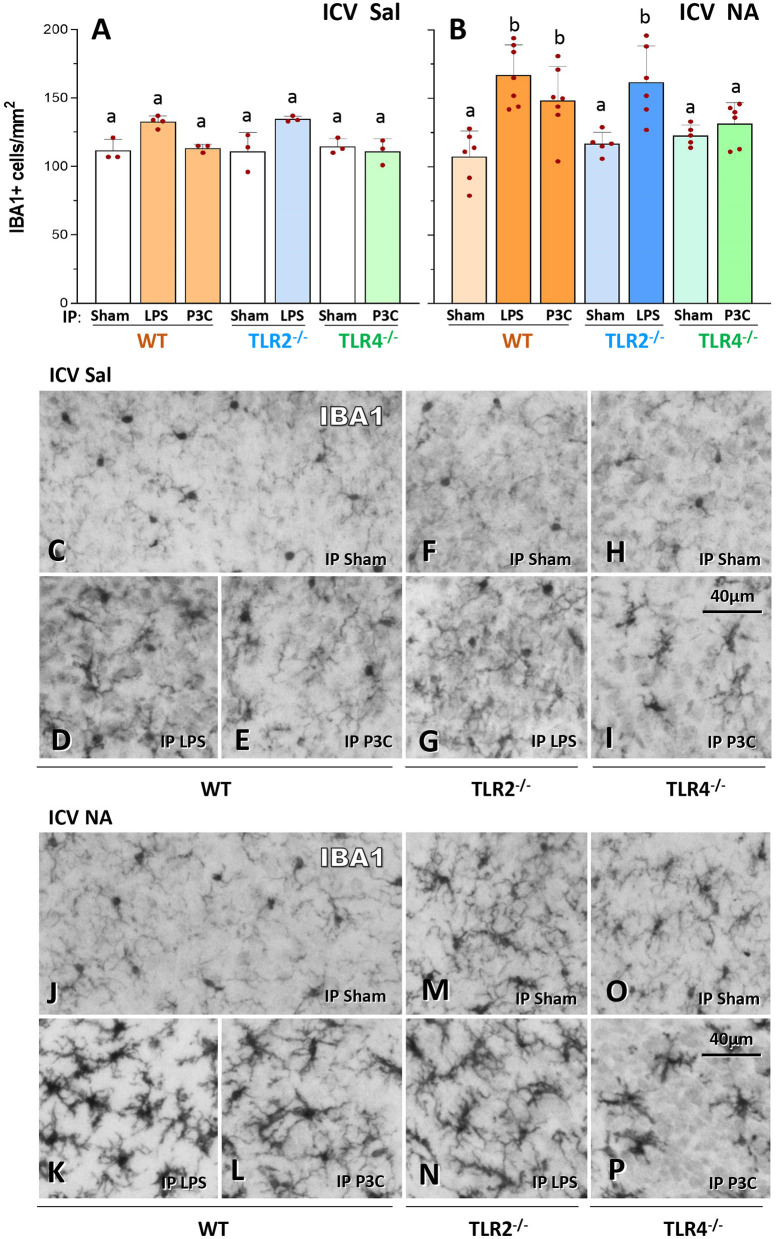
IBA1-positive cell counts in the hypothalamus of WT, TLR2^−/−^ and TLR4^−/−^ mice after a double inflammatory stimulus. IBA1-positive cells (microglia) were counted in the medio-dorsal periventricular region of the third ventricle **(A,B)**. Microglial density was higher in WT and TLR2^−/−^ animals that received two inflammatory stimuli [ICV NA and LPS or P3C, **(B)**] compared to control mice that received saline ICV [ICV Sal, **(A)**] or those injected IP with saline [ICV NA Sham in **(B)**]. On the contrary, in TLR4^−/−^ mice treated with double stimulus the number of IBA1-positive cells did not increase [ICV NA and P3C, **(B)**] compared to the same strain with only one stimulus (P3C in panel **A** and Sham in panel **B**). Representative images of IBA1 labeled sections from mice treated ICV with saline **(C–I)** or with NA **(J–P)**. In WT and TLR2^−/−^ mice that received the double stimulus **(K,L,N)** microglial cells appear more densely distributed and present a reactive profile (darker, with thicker and shorter ramifications), compared to the same strain with only one stimulus [**(D,E,J)** for WT; **(G,M)** for TLR2^−/−^]. Such difference is not observed in TLR4^−/−^ mice [**(P)** compared to **(I,O)**]. Bars in histograms **A** and **B** represent the mean ± s.d. of the plotted points [each point is the data of a single mouse; *n* = 3–4 in **(A)**, and *n* = 5–7 in **(B)**]. Means were compared by three-way ANOVA (*P* = 0.007). Letters a and b on top of the bars indicate the absence (if the same letter appears) or existence (if different letters appear) of a significant difference between the compared conditions, as indicated by Tukey's test (P < 0.05).

In order to investigate the maintenance of immune priming over time, a first inflammatory stimulus consisting of a single ICV injection of NA was applied to wild-type (WT) mice, and control mice were injected with saline. NA causes a transient sterile inflammation, which has been previously described in rats (Granados-Duran et al., 2015). To search for signs of immune training three months later, a second inflammatory stimulus was applied, in this case a peripheral stimulus consisting of an IP dose of LPS or P3C; control mice (Sham) were treated IP with saline (Figure 1). LPS and P3C were used because LPS activates the pattern receptor TLR4, while P3C is an agonist of TLR2. Thus, LPS was used as stimulus for TLR2^−/−^ mice, while P3C was used as stimulus for TLR4^−/−^ mice. One day after this second inflammatory threat, mice were sacrificed and microglia density was quantified by counting IBA1 positive cells (Figure 2). The brain region chosen for quantification was the medio-dorsal periventricular area of the third ventricle, as this area is known to be affected by the ventricular inflammation induced by NA injected ICV (Fernandez-Arjona et al., 2017, 2019b). WT mice exposed to both neuroinflammatory challenges (NA + LPS or NA + P3C) displayed higher microglial density compared to mice that received only the second one (compare bars WT-LPS and WT-P3C of Figure 2A with the same bars in Figure 2B). Not only the number of microglia was increased, but also the cells showed a more activated profile, evidenced by increased IBA1 label and thicker ramifications (compare Figures 2D,E with K,L respectively). The same pattern was observed in TLR2 mutant mice: upon the second IP-LPS stimulus, mice that received ICV NA displayed higher microglia density than those that received ICV saline (compare bar TLR2^−/−^-LPS in Figure 2A with the same bar in Figure 2B). Also, microglia appeared more activated (compare Figure 2G with Figure 2N). However, when TLR4 mutant mice were IP injected with P3C, those previously treated with NA exhibited a microglial density similar to mice ICV injected with saline (compare bar TLR4^−/−^-P3C in Figure 2A with the same bar in Figure 2B). In TLR4^−/−^ mice there was a slight morphological change of microglial cells after P3C stimulation (compare Figure 2I with Figure 2P) but much milder than that observed in WT mice (compare Figure 2L with Figure 2P). Finally, no differences were observed between the three strains of mice (WT, TLR2 and TLR4) when injected with saline IP (Sham), whether they had been treated ICV with NA or with saline (compare Figures 2A,B, as well as Figures 2C,F,H with Figures 2J,M,O).

These results evidence that, conversely to WT and TLR2^−/−^ mice, in TLR4^−/−^ mice microglial response to a second inflammatory stimulus is blunted, what suggests the absence of immune training in this strain.

### Higher density of astrocytes in WT and TLR2^−/−^ mutant mice, but not in TLR4^−/−^ mice, after a second inflammatory stimulus applied 3 months later

**Figure 3:**
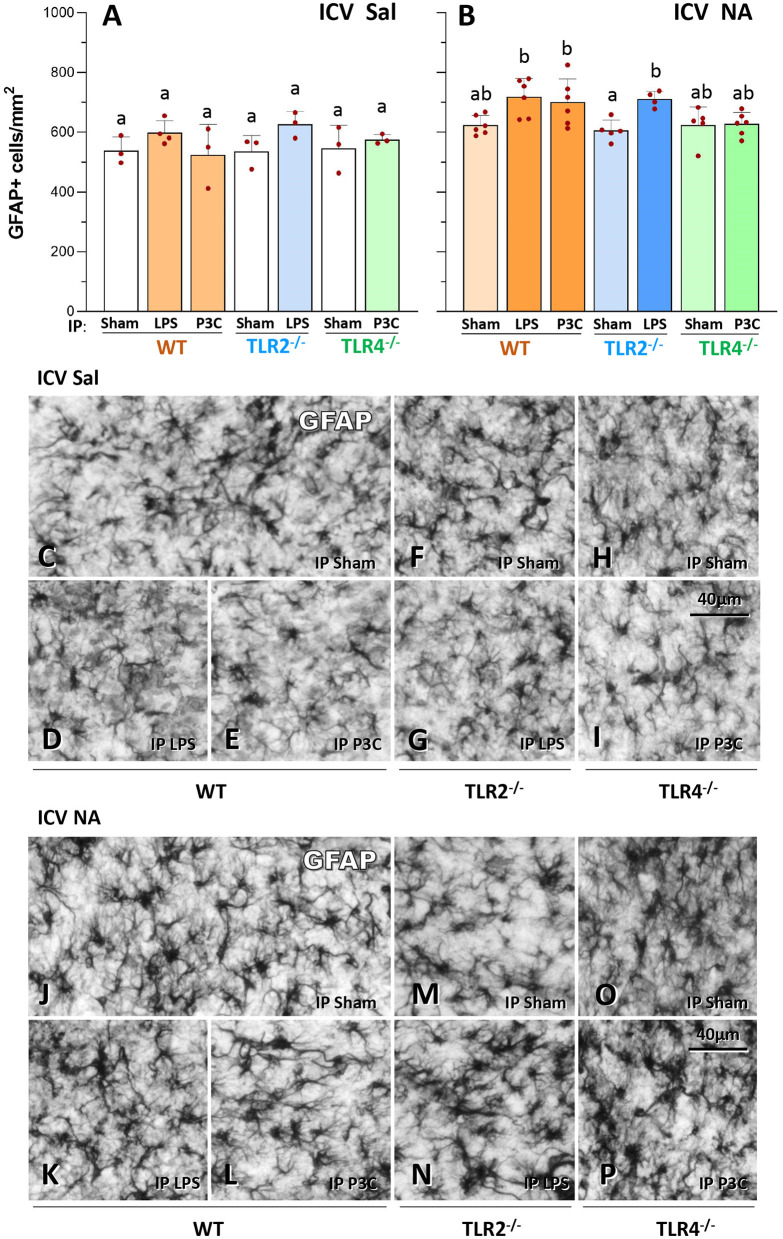
GFAP-positive cell counts in the hypothalamus of WT, TLR2^−/−^ and TLR4^−/−^ mice after a double inflammatory stimulus. The number of GFAP-positive cells (astrocytes) was evaluated in the periventricular region of the hypothalamus (medio-dorsal wall of the third ventricle). WT and TLR2^−/−^ mice that received a double inflammatory stimulus [NA ICV and LPS or P3C IP, **(B)**] had increased astrocytes density compared to animals of the same genotype receiving a single inflammatory stimulus [LPS/P3C in **(A)** or Sham groups in **(B)**]. However, TLR4^−/−^ mice displayed similar numbers of astrocytes in mice treated with two stimuli [NA ICV and P3C, **(B)**] and in those treated with a single one [P3C in **(A)** and Sham in **(B)**]. Micrographs show representative images of GFAP labeled sections from ICV Saline **(C–I)** and ICV NA **(J–P)** animals. Bars in histograms represent the mean ± s.d. of the plotted points [each point represents data from one mouse; *n* = 3–4 in **(A)** and *n* = 5–7 in **(B)**]. Means were compared by three-way ANOVA (P = 0.007). Letters a and b on top of the bars designate the absence (if the same letter appears) or existence (if different letters appear) of a significant difference between the compared groups, carried out by Tukey's test (*P* < 0.05).

GFAP-positive cell counts were carried out in a similar manner as explained in the previous section for IBA1. In WT and TLR2^−/−^ mice, a double inflammatory stimulus (NA + LPS or NA + P3C) provoked an increase in the density of astrocytes, compared to that observed in groups in which the first inflammatory stimulus was omitted (compare bars WT-LPS, WT-P3C and TLR2^−/−^-LPS of Figure 3A with the same bars of Figure 3B; and compare Figures 3D,E,G with Figures 3K,L,N respectively). On the contrary, in TLR4^−/−^ mutant mice IP challenged with P3C astroglial density was similar in mice previously treated ICV with NA or with saline (compare bar TLR4^−/−^-P3C in Figure 3A with the same bar in Figure 3B; and compare Figure 3I with Figure 3P). Thus, astrogliosis resulting from double stimulation in WT and TLR2^−/−^ mice, is however prevented in TLR4^−/−^ mice. Even though astrocyte density in TLR4^−/−^-P3C group (bar TLR4^−/−^-P3C in Figure 3B) was lower than that observed in WT-LPS, WT-P3C and TLR2^−/−^-LPS groups (bars WT-LPS, WT-P3C and TLR2^−/−^-LPS in Figure 3B) it was not statistically different (same letter b on all these bars), which suggests that astroglial density is not so much affected by the priming stimulus (NA ICV).

As previously observed for IBA1+ cell counts, no significant differences were found in GFAP+ cells between mice strains (WT, TLR2^−/−^ and TLR4^−/−^) when injected IP with saline (Sham), irrespective of the first stimulus (ICV NA or ICV Sal) applied (compare Figures 3A,B, as well as Figures 3C,F,H with Figures 3J,M,O).

Finally, the density of GFAP+ cells was similar in all groups that did not receive the second immune challenge (all IP Sham groups), irrespective of both the primary ICV stimulus (NA or Sal) and the mouse strain (compare Figures 3A,B, as well as Figures 3C,F,H with Figures 3J,M,O).

In conclusion, upon a second immune stimulus (LPS or P3C), a moderate increase in astrocytic density occurs in WT and TLR2^−/−^ mice, which is however not observed in TLR4^−/−^ mice, supporting the contribution of TLR4 to the acquisition of the immune training status.

### Gene expression analysis reveals moderate signs of inflammation and immune priming in the hypothalamus 3 months after NA-induced inflammation

The expression of a set of inflammation-related genes was examined in the hypothalamus of mice exposed to two inflammatory stimuli applied 3 months apart, specifically TNFα, IL-1β (two canonical pro-inflammatory cytokines), galectin 3 (Gal-3, an endogenous lectin upregulated in inflammation), NLRP3 (a protein that is part of the inflammasome 3 complex) and MHCII (a membrane protein upregulated in reactive and/or primed microglia).

**Figure 4:**
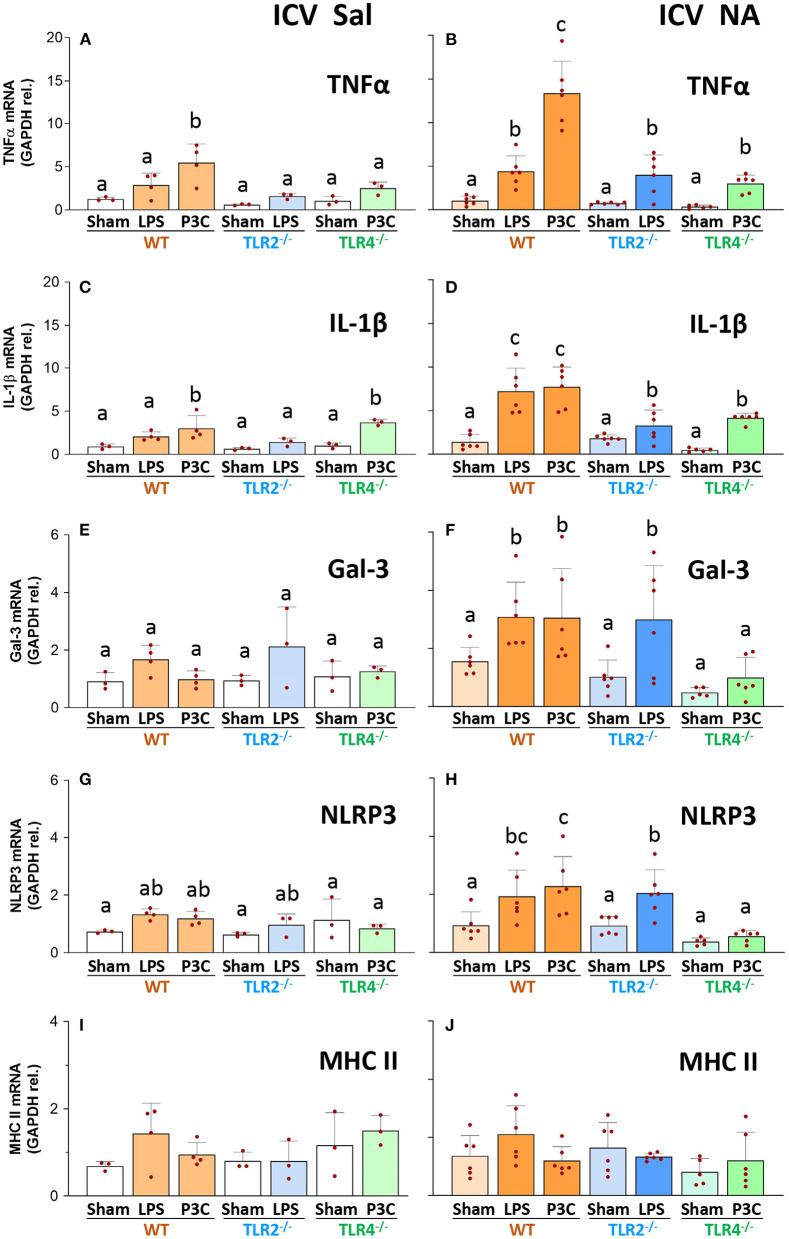
Gene expression of inflammatory markers in the hypothalamus of WT, TLR2^−/−^ and TLR4^−/−^ mice after a double inflammatory stimulus. The mRNA was extracted from the hypothalamus of mice first treated ICV with NA (controls injected with saline), and three months later stimulated again IP with LPS or P3C (Sham controls were IP injected with saline). The expression of TNFα, IL-1β, Gal-3, NLRP3 and MHC II was determined by qPCR, and normalized to the expression of GAPDH. In WT and TLR2^−/−^ animals that received the two stimuli the expression levels of TNFα, IL-1β and Gal-3 were increased [ICV NA and P3C or LPS, in **(B,D,F)**] compared to mice of the same strain with a single stimulus [ICV Sal in **(A,C,E)** and Sham groups in **(B,D,F)**]. The same tendency occurred for NLRP3, although the increase was milder **(G,H)**. Conversely, in TLR4^−/−^ mice the second stimulus was only able to induce a slight increase in the expression of TNFα **(B)** and IL-1β [**(D)** although not significant compared to ICV Sal in **(A)**], and for both genes such increase was to a lesser extent to that observed in WT [WT-P3C in **(B,D)**]. The expression of MHC II did not change significantly in any experimental group **(I,J)**. Bars in histogram represent the mean ± s.d. of the plotted points, each of which obtained from a different mouse [*n* = 3–4 in **(A,C,E,G,I)**; *n* = 5–7 in **(B,D,F,H,J)**]. Means were compared by three-way ANOVA (*P* = 0.007). Letters a, b and c on top of the bars point to the absence (if the same letter appears) or existence (if different letters appear) of a significant difference between the compared groups, carried out by the Tukey's test (*P* < 0.05).

The overall behavior of the expression levels of TNFα, IL-1β, Gal-3 and NLRP3 was quite similar (with minor differences) in the different experimental groups. Thus, expression levels were low in all groups and mouse strains that did not receive the first inflammatory stimulus (ICV-Saline groups; Figures 4A,C,E,G,I). Only in some cases the expression level slightly increased upon LPS or P3C injection (compare bar WT-P3C with the rest of bars in Figure 4A; compare bars WT-P3C and TLR4^−/−^-P3C with the rest of bars in Figure 4C), confirming that the IP immune stimulus was in fact provoking an immune response in the CNS.

However, when observing those groups treated ICV with NA and subjected to a double inflammatory stimulus (Figures 4B,D,F,H,J), WT mice that received LPS or P3C as second stimulus showed increased expression levels of TNFα, IL-1β and Gal-3 compared to their ICV-Saline counterparts (compare WT-LPS and WT-P3C bars in Figures 4B,D,F with the same bars in Figures 4A,C,E respectively). Similarly, the expression of NLRP3 was increased in the group WT-P3C previously treated ICV with NA compared to the same group injected ICV with saline (compare bar WT-P3C in Figure 4H with the same bar in Figure 4G). These results show that WT mice immunologically challenged 3 months after suffering an NA-induced inflammation process develop an enhanced response, suggesting a long-term immune priming in the nervous tissue.

Concerning TLR2^−/−^ mice that received the priming stimulus (ICV-NA), the expression levels of TNFα, IL-1β, and Gal-3 increased upon the second stimulus (LPS) compared with the same LPS treatment in the ICV-Sal group (compare bar TLR2^−/−^-LPS in Figures 4B,D,F with the same bar in Figures 4A,C,E), indicating that TLR2^−/−^ mice seem to have an immune priming similar to that of WT mice. However, if we compare WT and TLR2^−/−^ mice subjected to the double stimulus (NA+LPS), while the TNFα and Gal-3 increases upon LPS are similar in both strains (compare bars WT-LPS and TLR2^−/−^-LPS in Figures 4B,F), IL-1β increase is milder in TLR2^−/−^ than in the WT strain (compare bars WT-LPS and TLR2^−/−^-LPS in Figure 4D), suggesting that in the TLR2^−/−^ strain immune priming might be slightly decreased.

On the other hand, in TLR4^−/−^ mice the response upon the second stimulus (P3C) was similar in mice pre-treated with NA and those injected with saline, at least for the expression of IL-1β, Gal-3 and NLRP3 (compare bars TLR4-P3C in Figures 4D,F,H with the same bars in Figures 4C,E,G). In addition, when comparing the response to the double stimulus (NA+P3C) in TLR4^−/−^ mice with that of the WT strain, the expression levels of TNFα, IL-1β, Gal-3 and NLRP3 were consistently lower in the mutant strain (compare bars TLR4^−/−^-P3C with WT-P3C in Figures 4B,D,F,H). Therefore, when mice suffer a previous NA-induced inflammation, the inflammatory response of TLR4^−/−^ mice to a second stimulus (IP-LPS) is greatly diminished compared to that observed in WT mice. Conversely, the response in TLR2^−/−^ mice is very similar to that of the WT strain. This suggests that immune priming might be dependent, at least partially, on TLR4 signaling.

Finally, although MHC II is considered a marker of primed and/or reactive microglia, its expression levels did not change in any of the experimental groups (compare bars in Figures 4I,J).

When comparing the response induced by LPS and P3C in WT mice, in some cases P3C apparently elicited a heightened response than that of LPS (Figures 4A–C). The doses of LPS (300 μg/kg) and P3C (5 mg/kg) used here were based on those published by other authors, but we consider them not to be comparable in terms of the response they may provoke, as LPS (a highly purified fraction of lipopolysaccharide from *E. coli*) and P3C (a synthetic triacylated lipoprotein) are completely different compounds, and the doses used most probably not equivalent.

### Analysis of the interaction between factors on inflammation parameters

Three-way ANOVA revealed interactions between the factors examined in this experimental design, namely “ICV injection” (NA/saline), “IP injection” (LPS / P3C / saline) and “genotype” (TLR2^−/−^ / TLR4^−/−^ / WT).

First, the factors were evaluated individually. “ICV injection” and “IP injection” had effects on all parameters but MHC II expression, while “genotype” had an influence only in three of the measured parameters (Table 2, rows 1-3).

**Table 2:** Results of three-way ANOVA to analyze the interactions between the factors “ICV injection” (NA / saline), “IP injection” (LPS / P3C / saline) and “genotype” (TLR2^−/−^ / TLR4^−/−^ / WT) on the inflammatory parameters measured.

		**Parameters**						
		**Cell counts**		**Gene expression (qPCR)**				
		**IBA1+**	**GFAP+**	**TNFα**	**IL-1β**	**Gal-3**	**NLRP3**	**MHC II**
1	ICV injection (ICV)	*F*_1,64_ = 58.8 *P* < 0.001	*F*_1,64_ = 91.4 *P* < 0.001	*F*_1,63_ = 15.2 *P* < 0.001	*F*_1,63_ = 27.6 *P* < 0.001	*F*_1,63_ = 4.54 *P =* 0.038	*F*_1,63_ = 4.68 *P =* 0.035	*ns*
2	IP injection (IP)	*F*_2,64_ = 50.3 *P* < 0.001	*F*_2,64_ = 17.8 *P* < 0.001	*F*_2,63_ = 46.3*P* < 0.001	*F*_2,63_ = 32.1 *P* < 0.001	*F*_2,63_ = 7.77 *P =* 0.001	*F*_2,63_ = 6.67 *P =* 0.003	*ns*
3	Genotype (G)	*ns*	*ns*	*F*_2,63_ = 19.1*P* < 0.001	*F*_2,63_ = 5.40 *P =* 0.008	*ns*	*F*_2,63_ = 3.98 *P =* 0.025	*ns*
4	ICV*IP interaction	*F*_2,64_ = 12.5 *P* < 0.001	*ns*	*F*_2,63_ = 10.3 *P* < 0.001	*F*_2,63_ = 4.77 *P =* 0.013	*ns*	*ns*	*ns*
5	IP*G interaction	*F*_2,64_ = 4.94 *P =* 0.010	*ns*	*F*_2,63_ = 15.1 *P* < 0.001	*F*_2,63_ = 3.34 *P =* 0.044	*ns*	*ns*	*ns*
6	ICV*G interaction	*ns*	*F*_2,64_ = 2.94 *P =* 0.049	*F*_2,63_ = 5.84 *P =* 0.006	*F*_2,63_ = 6.07 *P =* 0.004	*F*_2,63_ = 3.42 *P =* 0.041	*F*_2,63_ = 5.22 *P =* 0.009	*ns*
7	ICV*IP*G interaction	*F*_2,64_ = 3.24 *P =* 0.045	*F*_2,64_ = 3.88 *P =* 0.025	*F*_2,63_ = 5.08 *P =* 0.010	*ns*	*ns*	*ns*	*ns*

ns, not significant.

When evaluating the interaction between factors, the interaction of “ICV injection” and “IP injection” (Table 2, row 4), as well as that of “IP injection” and “genotype” (Table 2, row 5), had an effect on IBA1+ cell counts and on the expression of TNFα and IL-1β. Besides, the interaction of “ICV injection” and “genotype” influenced the results of all parameters but IBA1+ cell counts and MHC II expression (Table 2, row 6). “ICV injection” and “IP injection” interaction (Table 2, row 4) reflects the enhanced effect of LPS/P3C on inflammatory parameters when injected to NA-treated mice, regardless the genotype. “IP injection” and “genotype” interaction (Table 2, row 5) highlights the differential impact of the IP injection of LPS/P3C in the different mouse strains used, whichever the previous ICV treatment. “ICV injection” and “genotype” interaction (Table 2, row 6) shows that the response to the ICV treatment differs between the different strains used, regardless the IP treatment.

Finally, a significant interaction between the three factors was found in the results obtained for IBA1+ and GFAP+ cell counts and on the expression of TNFα (Table 2, row 7). This relevant interaction reflects that the response to the ICV and IP treatments of at least one strain is different to that of the other strains, thus evidencing the blunted immune response upon de second stimulus of the TLR4^−/−^ strain, as the above exposed results have shown.

## Discussion

This work explores two issues related to immune priming in the brain: first, its maintenance over time, and second, the importance of the pattern receptors TLR2 and TLR4 in such process. A paradigm of two inflammatory stimuli was used to address these issues. The first one, consisting in an ICV injection of NA, which provokes an acute transient inflammation, aimed to induce the immune priming status. The second one, which was a peripheral inflammation provoked by LPS/P3C, pretended to show up such immune training. This second stimulus was applied three months after the first one, in order to investigate the long-lasting feature of immune priming. To evaluate the intensity of the immune response upon the second stimulus, the density of microglia and astrocytes was measured in the medio-dorsal periventricular region of the hypothalamus. This region was chosen because we know it is affected by the ventricular inflammation induced by NA (Granados-Duran et al., 2015; Fernandez-Arjona et al., 2017, 2019a,b). The expression of a panel of genes related to neuroinflammation was also examined in the hypothalamus.

### Immune priming in the brain after an acute episode of inflammation triggered by microbial neuraminidase

Increased numbers of microglia and astrocytes in the nervous parenchyma occur during immune responses in the brain (Bernaus et al., 2020; Muzio et al., 2021). In fact, using the same model of ICV injection of NA in rats, we reported increased microglial density in the hypothalamus 24 h after NA administration (Fernandez-Arjona et al., 2019a). However, according to our present results, 3 months after the ICV injection microglia population was similar in NA and saline injected mice (compare WT-Sham in Figures 2A,B). Thus, microglial density declines to normal levels over time, so it is a reversible feature of inflammation. On the other hand, we observed increased cell density upon a second inflammatory stimulus (LPS/P3C) when WT mice were previously injected with NA, but not in control mice ICV treated with saline. Not only the number, but also microglial morphology was skewed toward an activated profile. The same result was found for astrocytes, although in this case the increase in cell density was milder. These increases in microglial and astrocytic populations only in NA treated mice are suggesting an enhanced immune response compared to saline controls. On the other hand, gene expression (TNFα, IL-1β, Gal-3 and NLRP3) was also increased in the hypothalamus of NA-treated WT mice upon LPS/P3C, compared to saline-injected mice, what further suggests that the immune response is increased in those mice that previously suffered NA-induced neuroinflammation. Thus, these results point that a previous neuroinflammatory process (in this case provoked by NA) induces a state of immune training in the brain. Moreover, such immune priming persists over time, as it is evidenced 3 months after the priming stimulus.

Although in WT mice P3C seemed to induce a heightened response compared to LPS, this could be explained by the doses used of each compound. Because LPS and P3C are very different in nature, and the doses used here were chosen according to other reports, the intensity of their responses are hardly comparable. On the other hand, because LPS and P3C were administered peripherally, their effect on the brain could not be direct, but mediated by other inflammatory mediators produced peripherally, thus making it challenging to compare the degree of the neuroinflammatory response provoked by LPS and P3C.

The main candidate to account for immune training in the brain are microglial cells, which are considered the pivotal exponent of the immune system within the brain (Norden et al., 2015; Niraula et al., 2017; Neher and Cunningham, 2019). Upon activation, that can be triggered by a variety of stimuli, microglial cells proliferate, migrate to the damaged area (thus increasing cell density) and adopt a phagocytic and pro-inflammatory phenotype, with the production of cytokines and other inflammatory mediators (TNFα, IL-1β and Gal-3 among others) (Burguillos et al., 2015; Rodriguez-Gomez et al., 2020); morphological changes upon activation of microglia are also quite notable (Fernandez-Arjona et al., 2017). In recent years evidences have built up about the priming (or training) of microglial cells, an alternative functional state of these cells which is characterized by enhanced sensitivity and increased responses to any subsequent stimulus (Neher and Cunningham, 2019). This process has attracted attention because primed microglia have been proposed to be responsible for neurodegeneration and behavioral disturbances, among other alterations (Perry and Holmes, 2014; Colonna and Butovsky, 2017; Bartels et al., 2020). For this reason, strategies to modulate or inhibit microglial activation are under investigation (Spangenberg et al., 2016; Muzio et al., 2021; Tarale and Alam, 2022). In fact, using a strategy of microglial depletion Wendeln and colleagues concluded that microglia are mostly responsible for immune priming in the brain (Wendeln et al., 2018). However, the possibility that astrocytes might also engage a primed phenotype should not be ruled out. Our results show that, similarly to microglia, the density of astrocytes was higher in mice exposed to a double immune stimulation (NA + LPS/P3C), although this increase was milder than that of microglial cells. While activation of astrocytes could be secondary to that of microglia (Facci et al., 2014; Kirkley et al., 2017), some authors have reported evidences of priming in astrocytes (Hennessy et al., 2015; Muccigrosso et al., 2016). Future experiments should aim to explore to what extent are both microglia and/or astrocytes responsible for immune priming in the brain.

### Brain immune priming after NA-induced inflammation is enduring

In the experimental paradigm designed in this work the two inflammatory stimuli were applied 3 months apart, with the aim of assessing the long-term maintenance of immune training. Some works report chronic activation of microglia months or even 1 year after sepsis or traumatic brain injury (Holmin and Mathiesen, 1999; Loane et al., 2014; Trzeciak et al., 2019), but such situation is not equivalent to the immune training or primed state explored here, where a return to a not-inflamed scenario usually happens. Most studies carried out to date focus on exploring shorter time frames, in most cases within 1 month after the first immune challenge (Purisai et al., 2007; Weber et al., 2015; Muccigrosso et al., 2016; Frank et al., 2020; Martins-Ferreira et al., 2021). Recently we reported microglial priming in the hypothalamus 3 months after the ICV administration of NA (Fernandez-Arjona et al., 2022). Furthermore, in accordance to our results, using an experimental design of immune training by peripheral injections of LPS, Wendeln and colleagues showed that immune priming in the brain lasted for at least 6 months (Wendeln et al., 2018). Therefore, immune priming in the brain seems to be a long enduring feature, that could eventually affect the outcome of new future insults.

In the present work, longer survival times could have been explored; however, because aging itself may trigger microglial priming (Godbout et al., 2005; Niraula et al., 2017) confounding results could be obtained. By the time of sacrifice the mice were 5 months of age, which is considered a young age for mice (Flurkey et al., 2007). Thus, the brain immune response observed after the second stimulus can be attributed to immune training, not dependent on age but on a previous immune stimulus.

### The receptor TLR4, but not TLR2, is essential for NA-driven immune priming

The other main question addressed in this work was the relevance of the pattern recognition receptors TLR2 and TLR4 in the acquisition of immune training. For this purpose, mutant mice for each of these receptors were used, along with WT mice, in our double-inflammatory stimulus experimental design. Evidences of the immune response (density of microglia and astrocytes; inflammation-related genes expression) were evaluated after the second IP stimulus, this being LPS (a ligand of TLR4) for TLR2 mutants and P3C (a ligand of TLR2) for TLR4 mutants. TLR2 mutant mice behaved in a similar manner as WT mice, that is, after P3C injection the number of microglia and astrocytes increased, as well as the expression of TNFα, IL-1β, Gal-3 and NLRP3. Therefore, TLR2 receptor does not seem to be necessary for achieving NA-driven immune training in the brain. However, the IL-1β response to LPS was milder than that observed in WT mice (compare WT-LPS and TLR2^−/−^-LPS in Figure 4D), suggesting a partial involvement of this receptor. Conversely, the response observed in TLR4 mice was completely different to that seen in WT mice. In TLR4 mutants treated with NA microglial and astrocytic density did not increase after P3C stimulation. The expression levels of Gal-3 and NLRP3 did not increase either, while the increase in the expression of TNFα and IL-1β was dampened compared to that of WT mice (compare WT-P3C and TLR4^−/−^-P3C in Figures 4B,D). These results point that immune priming is absent, or at least severely compromised, in TLR4 mutant mice, suggesting that TLR4 receptor is essential for the establishment of immune training in the brain, at least when the priming stimulus is NA. TLR2 receptor, while not being that relevant for immune priming establishment, seems to be partially involved. Other authors have reported evidences in the same line. When using a stressor as priming stimulus, increased levels of glucocorticoids stimulated the synthesis and release of the danger signal HMGB1, which acts through receptors TLR2 and TLR4 to induce priming of the inflammasome NLRP3, thus favoring immune memory and a heightened response to subsequent stimuli (Frank et al., 2015). Furthermore, blocking TLR2 and TLR4 signaling with OxPAPC (an antagonist of both receptors) prevented such stress-induced priming (Weber et al., 2015). Also, experiments *in vitro* demonstrated priming of microglia using TLR2, −3 and −4 specific ligands, and the importance of such priming for ATP-dependent IL-1β release. The same work reported that such TLR2 / −3 / −4 dependent priming did not occur in astrocytes (Facci et al., 2014). Further studies have been carried out with peripheral immune cells. In macrophages, immune tolerance was induced with specific ligands for different TLR receptors, but tolerance mediated by TLR4 activation with LPS was the archetype (Butcher et al., 2018). Conversely to immune training, immune tolerance refers to a process where responses to subsequent stimuli are suppressed, thus providing a limit to the potentially harmful consequences of sustained levels of pro-inflammatory cytokines (Butcher et al., 2018; Wendeln et al., 2018). In either version of immune memory, tolerance or training/priming, TLR receptors have been implicated (Weber et al., 2013; Facci et al., 2014; Braza et al., 2018; Butcher et al., 2018), and epigenetic programming has been suggested to be underneath the plasticity of the immune responses after repeated stimulations (Saeed et al., 2014; Cho et al., 2015; Shalova et al., 2015).

Interestingly, the response (in terms of gene expression, Figure 4) of TLR2^−/−^ mice to LPS was slightly diminished compared to that of WT mice. Similarly, the response of TLR4^−/−^ mice to P3C was mildly decreased compared to WT. In a previous work using cultured microglia isolated from WT, TLR2^−/−^ and TLR4^−/−^ mice which were *in vitro* challenged with LPS and P3C, we observed similar results (Fernandez-Arjona et al., 2019a). It has been reported that TLR receptors, and particularly TLR2 and TLR4, may interact and form heterodimers in the cell surface, thus cooperating with each other in their function (Klein et al., 2008; Rosenberger et al., 2014; Wang et al., 2014). We hypothesize that the lack of TLR2 might affect the responses mediated by TLR4 and, similarly, the responses due to TLR2 activation could be somehow influenced by the absence of TLR4 receptor, thus explaining the diminished responses mediated by one receptor when the other is missing.

On the other hand, it is opportune to point out that LPS and P3C simulate inflammatory responses induced by bacterial infections, but LPS/P3C-induced neuroinflammation might not be a universally adapted neuroinflammatory response.

Here we used neuraminidase (NA) as the priming stimulus. Various TLR ligands have been more widely used for this purpose (Facci et al., 2014). However, we have consistently used NA because 1) it is present in the envelope/cell wall of various neurotropic microbes such as influenza, mumps, meningitis, etc., contributing to their pathogenicity and/or dispersion (O'Toole et al., 1971; Love et al., 1985; Steininger et al., 2003; Finsterer and Hess, 2007; Yildizdas et al., 2011; Lewis and Lewis, 2012); and 2) NA provokes an acute inflammatory process, which has been well characterized (Granados-Duran et al., 2015; Fernandez-Arjona et al., 2017, 2019b). We previously showed that NA activates microglial cells mainly through TLR4, being its sialidase activity essential (Fernandez-Arjona et al., 2019a); we proposed a desialylation mechanism, rather than binding of NA as a ligand, for TLR4 activation by NA. However, in the inflammatory scenario arisen after an ICV injection of NA other inflammatory mediators and alarmins signaling through TLR4 may be present. Thus, in our experiment NA may not be the only factor inducing TLR4 activation during the priming stage. Nevertheless, comparisons with control animals ICV injected with saline help to highlight the importance of NA as the priming stimulus. In fact, our results point the existence of immune priming only in those animals treated with NA. Therefore, apparently the inflammation provoked by the ICV procedure does not seem enough to induce the immune training, while the presence of NA in the same scenario does. As NA is a component of neurotropic virus/bacteria (Love et al., 1985; Steininger et al., 2003; Yildizdas et al., 2011; Nishikawa et al., 2012; McAuley et al., 2019), these results highlight the potential consequences of brain infections by those pathogens on the immune training of the nervous tissue. Furthermore, sialidase inhibitors (which are presently used as treatment for infections such as influenza) could be considered to prevent immune priming in cases of NA-bearing pathogens.

## Conclusions

In summary, here we show the establishment of immune priming in the brain after an episode of acute inflammation triggered by a single ICV injection of NA. The presence of NA itself is required for such immune training, as it does not occur in saline injected mice. This is of interest because NA is beard by various pathogens able to provoke brain infections. On the other hand, the pattern receptor TLR4 is essential for immune priming driven by NA, while TLR2 seems to play a minor role. Moreover, immune memory is durable, as it stays for at least 3 months. Because immune priming results in enhanced subsequent inflammatory processes that may have deleterious consequences, past brain infections (in particular those provoked by NA-bearing pathogens) should be considered a risk factor in the outcome of future inflammatory events, even if these were peripheral.

## Summary statement

WT and TLR2 mutant mice develop long-term immune priming after acute neuroinflammation induced by microbial neuraminidase injected into the cerebral ventricles. Such immune priming is largely missing in TLR4 mutant mice. Thus, brain immune priming driven by microbial neuraminidase is broadly dependent on TLR4 receptor, while TLR2 has a minor role.

## Data availability statement

The raw data supporting the conclusions of this article will be made available by the authors, without undue reservation.

## Ethics statement

The animal study was reviewed and approved by Comité Ético de Experimentación de la Universidad de Málaga Reference 2012-0013. Animal care and handling were performed according to the guidelines established by Spanish legislation (RD 53/2013) and the European Union regulation (2010/63/EU).

## Author contributions

MF-A: methodology, formal analysis, writing—original draft, writing—review and editing, and visualization. AL-R: methodology. JG: conceptualization, methodology, writing—original draft, writing—review and editing, and funding acquisition. ML-A: conceptualization, writing—original draft, writing—review and editing, and funding acquisition. All authors contributed to the article and approved the submitted version.

## Funding

This work was supported by funding from Ministerio de Economía, Industria y Competitividad (Spanish Government; grant number SAF2017-83645). AL-R received fellowships from Plan Propio de Investigación y Transferencia (Universidad de Málaga, Spain). The Olympus VS120 microscope was acquired with FEDER funds from the European Union.

## Conflict of interest

The authors declare that the research was conducted in the absence of any commercial or financial relationships that could be construed as a potential conflict of interest.

## Publisher's note

All claims expressed in this article are solely those of the authors and do not necessarily represent those of their affiliated organizations, or those of the publisher, the editors and the reviewers. Any product that may be evaluated in this article, or claim that may be made by its manufacturer, is not guaranteed or endorsed by the publisher.
